# Dimension reduction of microbiome data linked *Bifidobacterium* and *Prevotella* to allergic rhinitis

**DOI:** 10.1038/s41598-024-57934-x

**Published:** 2024-04-05

**Authors:** Shohei Komaki, Yukari Sahoyama, Tsuyoshi Hachiya, Keita Koseki, Yusuke Ogata, Fumiaki Hamazato, Manabu Shiozawa, Tohru Nakagawa, Wataru Suda, Masahira Hattori, Eiryo Kawakami

**Affiliations:** 1 Genome Analytics Japan Inc., Tokyo, Japan; 2 Technology Strategy Div., Hitachi High-Tech Corporation, Business Tower, Toranomon Hills, 1-17-1 Minato-ku, Toranomon, Tokyo 105-6409 Japan; 3 Advanced Data Science Project (ADSP), RIKEN Information R&D and Strategy Headquarters, RIKEN, Yokohama City, Kanagawa 230-0045 Japan; 4 Laboratory for Microbiome Sciences, RIKEN Center for Integrative Medical Sciences, Yokohama, Japan; 5 Hitachi Health Care Center, Hitachi Ltd., Ibaraki, Japan; 6 Graduate School of Advanced Science and Engineering, Waseda University, Tokyo, Japan; 7 Artificial Intelligence Medicine, Graduate School of Medicine, Chiba University, Chiba City, Chiba 260-8670 Japan; 8 Institute for Advanced Academic Research (IAAR), Chiba University, Chiba City, Chiba 260-8670 Japan

**Keywords:** Clinical microbiology, Microbiome

## Abstract

Dimension reduction has been used to visualise the distribution of multidimensional microbiome data, but the composite variables calculated by the dimension reduction methods have not been widely used to investigate the relationship of the human gut microbiome with lifestyle and disease. In the present study, we applied several dimension reduction methods, including principal component analysis, principal coordinate analysis (PCoA), non-metric multidimensional scaling (NMDS), and non-negative matrix factorization, to a microbiome dataset from 186 subjects with symptoms of  allergic rhinitis (AR) and 106 controls. All the dimension reduction methods supported that the distribution of microbial data points appeared to be continuous rather than discrete. Comparison of the composite variables calculated from the different dimension reduction methods showed that the characteristics of the composite variables differed depending on the distance matrices and the dimension reduction methods. The first composite variables calculated from PCoA and NMDS with the UniFrac distance were strongly associated with AR (FDR adjusted *P* = 2.4 × 10^–4^ for PCoA and *P* = 2.8 × 10^–4^ for NMDS), and also with the relative abundance of *Bifidobacterium* and *Prevotella*. The abundance of *Bifidobacterium* was also linked to intake of several nutrients, including carbohydrate, saturated fat, and alcohol via composite variables. Notably, the association between the composite variables and AR was much stronger than the association between the relative abundance of individual genera and AR. Our results highlight the usefulness of the dimension reduction methods for investigating the association of microbial composition with lifestyle and disease in clinical research.

## Introduction

Allergic rhinitis (AR), a condition affecting over half a billion people worldwide^1^, is triggered by immunoglobulin E (IgE)-mediated responses to airborne allergens, resulting in symptoms such as nasal itching, sneezing, and congestion^2^. This condition not only reduces quality of life, but also contributes to reduced cognitive function and increased irritability^3^. AR is also associated with an increased risk of developing asthma^2^. The etiology of AR is still not fully understood, despite its recognition as a major health problem. Emerging research points to the gut microbiota as a key player in the development of allergies^4–6^. The human gut harbours a wide variety of microorganisms and the composition of the human microbiome varies from person to person. Dietary components can influence the gut microbiome and modulate allergic responses through the production of bioactive metabolites and their interactions with immune cells^4–7^.

In clinical research, enterotyping has been used as a method to characterise the microbial composition of the gut^7,8^. In a seminal paper defining enterotypes for the first time, the human gut microbiota was classified into three enterotypes: P-type (*Prevotella*-rich), B-type (*Bacteroides*-rich), and R-type (*Ruminococcus*-rich)^8^. Subsequent studies have shown that the composition of the human microbial community is not discretely distributed as enterotypes, but rather continuously distributed in typical populations^9,10^.

Dimension reduction methods such as principal component analysis (PCA) and principal coordinate analysis (PCoA) are commonly used to visualise the distribution of the human microbiome community^11,12^. Dimension reduction methods illustrate the distributions of microbial samples by mapping multidimensional data of microbial composition onto a few composite dimensions based on the distances (or dissimilarities) between samples. The composite variables calculated by the dimension reduction methods are robust to technical and biological noise. However, in clinical research, the composite variables calculated by dimension reduction methods have not been widely used to study the association of the human gut microbiome with lifestyle and disease.

There are several methods for calculating the dissimilarity between samples and obtaining composite variables from the distance matrix^12^. Here, we investigated the association of the human gut microbial composition with dietary intake of 42 nutrients and the symptom of AR, focusing on the application and comparison of several dimension reduction methods, including PCA, PCoA, non-metric multidimensional scaling (NMDS), and non-negative matrix factorization (NMF).

## Materials and methods

### Samples and datasets

In the present study, we re-analysed a microbiome dataset from 186 participants with symptoms of AR and 106 controls without symptoms of AR at the Hitachi Health Care Centre in Japan. The dataset was used in our previous study to identify up- and down-regulated microbial genera in AR patients compared to controls^13^. No dimension reduction method was used in the previous study. The present study investigated the association of the human gut microbial composition with dietary intake of 42 nutrients and the symptom of AR, focusing on the application and comparison of dimension reduction methods.

In brief, food consumption data for the study participants were obtained using the Brief Self-Administered Diet History Questionnaire (BDHQ) and adjusted by energy using the density method^14–16^. Bacterial DNA was isolated from faecal samples, followed by amplification of the 16S V1-V2 region by polymerase chain reaction (PCR)^17^. An equal amount of each PCR amplicon was mixed and subjected to multiplex amplicon sequencing using MiSeq (2 × 300 paired-end). Filtered reads with BLAST match lengths < 90% to the representative sequence in the 16S databases, including the Ribosomal Database Project (RDP) (Release 11, Update 5), CORE (updated 13 October 2017; http://microbiome.osu.edu/), and a reference genome sequence database obtained from the NCBI FTP site (ftp://ftp.ncbi.nih.gov/genbank/, April 2013), were considered chimeras and removed. From the filtered reads, 10,000 high quality reads per sample were randomly selected. The total reads were then sorted by the frequency of redundant sequences and grouped into operational taxonomic units (OTUs) using UCLUST with a sequence identity threshold of 97%. The representative sequences of the generated OTUs were subjected to a homology search against the above databases using the GLSEARCH program for taxonomic assignments. Phylum, genus and species level assignments were made using sequence similarity thresholds of 70%, 94% and 97%, respectively.

This study was approved by the Hitachi Hospital Group Ethics Committee (Approved No. 2018-5, 2019-10, and 2020-88), the Institutional Review Board of the Hitachi Ltd. (Approved No. 220-1 and 238-1), and the Research Ethics Committee (Approved No. H30-5). Written informed consent was obtained from all participants. This study was conducted in accordance with the principles of the Declaration of Helsinki.

### Dimension reduction of microbial composition

Genus-level abundance was expressed as a percentage. Genera with a mean relative abundance of ≥ 0.1% were included, resulting in a genus-level abundance matrix with 292 rows (samples) and 50 columns (genera).

We first performed enterotyping to classify the microbiome of 292 individuals as in the landmark study^8^. Following the enterotyping R tutorial (https://enterotype.embl.de/), the Jensen-Shannon divergence (square root of the Jensen-Shannon distance; JSD) between all pairs of 292 samples was calculated and a 292 × 292 pairwise distance matrix was generated. The JSD is a symmetrized and smoothed version of the Kullback–Leibler divergence which measures the similarity of the probability distributions of two samples^18^. Based on the distance matrix, 292 samples were then clustered into the discrete enterotypes by the partitioning around medoids algorithm using the clusterSim R package (version 0.50.1)^19^. The number of clusters was set at 3 as in the seminal study^8^.

We also used several dimension reduction methods, including PCA, PCoA, NMDS, and NMF, to obtain the continuous composite variables from the above-mentioned genus-level abundance matrix of 292 samples and 50 genera. The top 3 composite variables for each dimension reduction method were obtained for subsequent analyses.

To perform the PCA, we used the prcomp function implemented in the stats R package (version 4.2.1)^20^ with the scale = TRUE option, which normalises the genus-level abundance matrix so that each column has a mean of 0 and a standard deviation of 1. In addition, PCA was performed on the centered log-ratio (CLR) transformed abundance matrix. For the CLR transformation, the clr function implemented in the compositions R package (version 2.0.6)^21^ was used.

To perform the PCoA, we applied the cmdscale function in the stats R package (version 4.2.1) to the above-mentioned JSD to obtain the top 3 composite variables. In addition, we calculated the Bray–Curtis dissimilarity (BCD) matrix^22^ using the beta.pair.abund function implemented in the betapart R package, which measures the compositional difference between two ecological communities (version 1.5.6)^23^ with “bray” specified as index.family, and the weighted UniFrac distance matrix^24^, which takes into account the phylogenetic distance between genera, using USEARCH (version 10.0.240_i86)^25^. The cmdscale function was applied to the BCD and UniFrac matrices to obtain the top 3 composite variables.

For the NMDS analysis, the metaMDS function from the vegan R package was used to obtain the top 3 composite variables from JSD, BCD, and UniFrac distance matrices. Prior to generating the JSD and BCD matrices, the microbiome abundance matrix was standardized and multiplied by the total sample size using the decostand function from the vegan R package (version 2.6.2)^26^.

Non-negative matrix factorization is a dimension reduction method that decomposes a non-negative matrix V into two non-negative matrices W and H, such that V is approximately equal to W multiplied by H^27^. The matrix W represents the composite variables, while H represents the coefficients when the original data are expressed as a linear combination of the composite variables. The number of composite variables is set as 3. The reconstruction error between V and WH is minimised by iteratively updating W and H according to some loss function. We used the NNLM R package (version 0.4.4)^28^ to apply NMF to the genus-level abundance matrix V. We used the Kullback–Leibler divergence as the loss function to minimise the reconstruction error.

For comparison, we also calculated the ratio of *Prevotella* to *Bacteroides* (P/B ratio)^29,30^.

### Statistical analysis

To examine the similarity between composite variables, and to examine the association between composite variables and genus-level abundance, we applied the pairwise Spearman's rank correlation test using the cor function from the stats R package with the “spearman” method. We evaluated the association of composite variables with the intake levels of 42 nutrients using the Spearman's rank correlation test. The association of composite variables with AR was tested by the Wilcoxon-Mann–Whitney test using the wilcox_test function from the coin R package (version 1.4.2)^31^, with the distribution set to “exact”. For each dimension reduction method, we calculated the top three composite variables and compared them with 50 genera (3 × 50 = 150 tests), 42 nutrients (3 × 42 = 126 tests) and allergic rhinitis (3 × 1 = 3 tests). We applied the Benjamini and Hochberg false discovery rate (FDR)^32^ correction for multiple testing to the sets of 150, 126 and 3 *P* values, respectively, using the p.adjust function in the stats R package, specifying “BH” as method. To investigate the potential impact of confounders, we also assessed the association between each composite variable (an explanatory variable) and AR (an outcome variable) using logistic regression adjusted for age, sex, and BMI. The outcome variable of AR was further regressed on the microbiome distance matrix by the microbiome regression-based kernel association test (MiRKAT) using the MiRKAT R package (version 1.2.3)^33^. We used three 292 × 292 pairwise distance matrices (JSD, BCD, and UniFrac) for the MiRKAT analysis.

## Results

### Continuous distribution of gut microbial community

**Figure 1:**
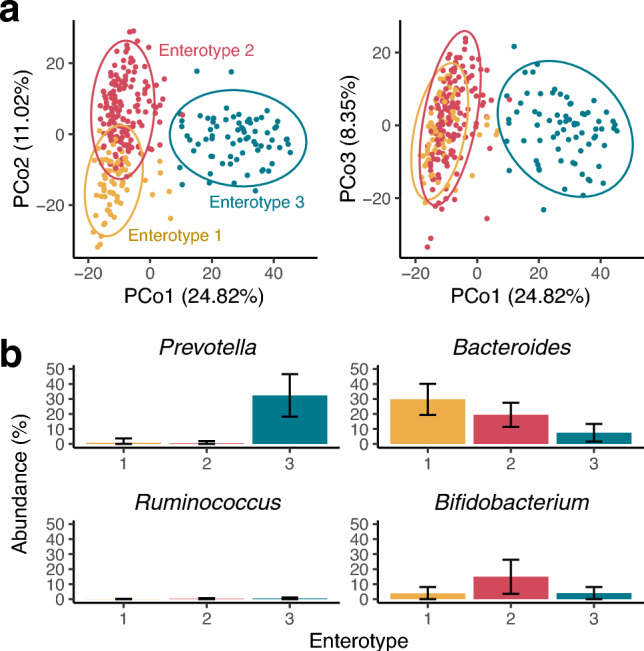
Enterotypes and distribution of gut microbial composition. (**a**) Principal coordinate analysis plots based on the Jensen-Shannon divergence. The left panel shows the top 1 (x-axis) and 2 (y-axis) composite variables, while the right panel shows the top 1 (x-axis) and 3 (y-axis) composite variables. The dot colour indicates the enterotype calculated from the same Jensen-Shannon divergence matrix. (**b**) Relative abundances of representative genera by enterotype.

**Figure 2:**
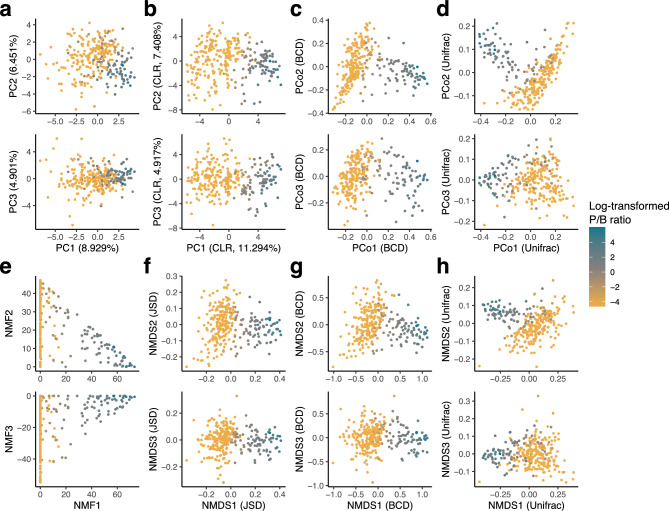
Distribution of the gut microbial community using different dimension reduction methods. (**a**) principal component analysis (PCA), (**b**) PCA on the centered log-ratio (CLR) transformed abundance matrix, (**c**, **d**) principal coordinate analysis with the Bray–Curtis dissimilarity (BCD) and the UniFrac distance, (**e**) non-negative matrix factorization, (**f**–**h**) non-metric multidimensional scaling with the Jensen-Shannon distance, BCD, and UniFrac distance. The top panel shows the top 1 (x-axis) and 2 (y-axis) composite variables, while the bottom panel shows the top 1 (x-axis) and 3 (y-axis) composite variables. The colour of the point indicates the log-transformed P/B ratio.

There were 50 genera with mean relative abundance ≥ 0.1%. The JSD matrix was calculated from the 50-dimensional genus-level data of the 292 individuals. Enterotypes were calculated from the JSD matrix (Fig. 1a). *Bacteroides* and *Prevotella* were abundant in the enterotype 1 (corresponding to B-type) and 3 (P-type), respectively (Fig. 1b; Table 1). The abundance of *Ruminococcus* was low across all enterotypes, whereas *Bifidobacterium* was abundant in the enterotype 2 (Fig. 1b; Table 1). The distributions of enterotypes 1 and 2 overlapped in the PCoA plots, emphasising that the microbial composition was continuous rather than discrete. The continuous distribution of gut microbial composition was further supported by other dimension reduction methods, including PCA (Fig. 2a,b), PCoA with the BCD and UniFrac (Fig. 2c,d), NMF (Fig. 2e), and NMDS with the JSD, BCD, and UniFrac (Fig. 2f–h).

**Table 1:** Characteristics of the study participants.

	Enterotype 1	Enterotype 2	Enterotype 3
n (woman)	71 (6)	149 (22)	72 (4)
Age, year	50.5 ± 7	49.1 ± 7.9	49.9 ± 7.4
BMI, kg/m2	23.7 ± 3.1	23.3 ± 3.2	24.1 ± 2.6
Allergic rhinitis, %	64.8	69.8	50
*Prevotella*, %	0.8 ± 2.9	0.4 ± 1.6	32.4 ± 14.2
*Bacteroides*, %	29.8 ± 10.4	19.4 ± 8.1	7.5 ± 5.8
*Ruminococcus*, %	0 ± 0.2	0.2 ± 0.5	0.4 ± 0.7
*Bifidobacterium*, %	3.8 ± 4.2	14.9 ± 11.4	4 ± 4.1

### Comparison of composite variables calculated using different dimension reduction methods

**Figure 3:**
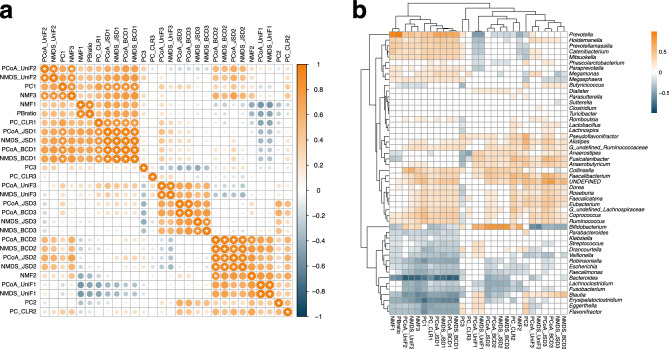
Comparison and characterisation of composite variables. (**a**) Pairwise Spearman's rank correlations between composite variables. X- and y-axes were ordered based on hierarchical clustering. Highly correlated pairs (Spearman's rank correlation > 0.8) are indicated by asterisks (*). (**b**) Spearman's rank correlations between composite variable and genus-level relative abundance. Non-significant pairs (FDR-adjusted *P* value > 0.05) are shown as white cells. NMF: non-negative matrix factorization, P/B ratio: *Prevotella*-*Bacteroides* ratio, PC: principal component, CLR: centered log-ratio transformed, PCo: principal coordinate, NMDS: non-metric multidimensional scaling, JSD: Jensen-Shannon divergence, BCD: Bray–Curtis dissimilarity, UniF: UniFrac distance.

To compare the composite variables obtained from the different dimension reduction methods, we calculated the Spearman's rank correlation between pairs of composite variables (Fig. 3a). The results showed that the top 3 composite variables calculated from the PCoA and NMDS using the JSD were highly correlated with those calculated from the PCoA and NMDS using the BCD. The top 3 composite variables calculated from the PCoA using the UniFrac distance were highly correlated with those calculated from NMDS using the UniFrac distance. The top 1 composite variable calculated from PCA was highly correlated with those from PCoA and NMDS, while the second and third composite variables from PCA were not remarkably correlated with those calculated from the other methods. The first composite variable calculated by NMF was highly correlated with the P/B ratio, second composite variable was correlated with those of PCoA and NMDS using JSD and BCD, and the third composite variable was highly correlated with the first principal component and the second composite variables of PCoA and NMDS using the UniFrac distance.

The first composite variable calculated using PCoA and NMDS with JSD and BCD was positively correlated with the relative abundance of *Prevotella* and negatively correlated with *Bacteroides* (Fig. 3b). The second composite variable calculated using PCoA and NMDS was positively correlated with *Bifidobacterium*, while the third composite variable was negatively correlated with *Bifidobacterium*. The first composite variable calculated using PCoA and NMDS with the UniFrac distance was positively correlated with the relative abundance of *Bifidobacterium* and negatively correlated with *Prevotella*. The second composite variable was negatively correlated with *Bacteroides*, while the third composite variable was negatively correlated with *Bifidobacterium*. The first composite variable calculated using NMF was positively correlated with *Prevotella* and negatively correlated with *Bacteroides*, as was the P/B ratio.

### Nutrients and AR were related to microbial composition via composite variables

We examined the association of composite variables calculated using different dimension reduction methods with the consumption levels of 42 nutrients and AR. The MiRKAT analysis, which tested the association of distance matrices rather than composite variables with AR, showed that the UniFrac distance was significantly associated with AR (*P* = 3.7 × 10^–4^), whereas the JSD and BCD distances were not (*P* > 0.05). No significant association was observed between the microbiome distance matrices and nutrient intakes.

**Figure 4:**
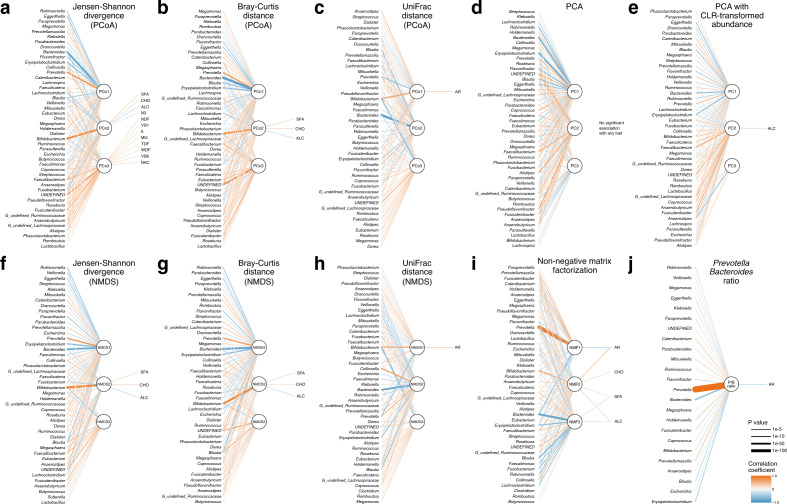
Associations of microbial composition with nutrient intakes and AR via composite variables. Circles indicate the top 3 composite variables calculated using different dimension reduction methods. Significant associations (FDR adjusted *P* value < 0.05) of the composite variables with genus-level abundance, nutrient intake, and the symptom of AR are shown. The line thickness represents -log10(*P* value) and the line color indicates the direction of association (coefficient). Orange and blue line indicate positive and negative associations, respectively. (**a**–**c**) Principal coordinate analysis (PCoA) with the Jensen-Shannon divergence (JSD) the Bray–Curtis dissimilarity (BCD), and the UniFrac distance; (**d**) principal component analysis (PCA); (**e**) PCA with the centered log-ratio (CLR) transformed abundance matrix; (**f**–**h**) non-metric multidimensional scaling (NMDS) with the JSD, BCD, and the UniFrac distance; (**i**) non-negative matrix factorization, and (j) *Prevotella* to *Bacteroides* ratio. ALC: alcohol, CHO: carbohydrate, K: potassium, MG: magnesium, NAC: niacin, NDF: insoluble dietary fibre, SFA: saturated fat, TDF: total dietary fibre, VB1: thiamin, VB6: vitamin B6, WDF: soluble dietary fibre.

According to the Wilcoxon-Mann–Whitney test, AR was associated with the first composite variable calculated from the UniFrac distance (FDR adjusted *P* = 2.4 × 10^–4^ for PCoA and *P* = 2.8 × 10^–4^ for NMDS), the first and second composite variables calculated from the NMF (FDR adjusted *P* = 0.0048 and 0.029, respectively), and the P/B ratio (*P* = 0.013; Fig. 4j), while the other composite variables were not significantly associated with AR. Logistic regression analysis adjusted for age, sex, and BMI also confirmed that the composite variables significantly associated with AR in the Wilcoxon-Mann–Whitney test were significantly associated with AR (Supplementary Table S1). The first composite variables calculated from the UniFrac distance (PCoA and NMDS), which showed the strongest positive association with AR, were significantly associated with increased abundance of *Bifidobacterium* and decreased abundance of *Prevotella* (Fig. 4c,h). Notably, the association between these composite variables and AR was stronger than the association between the relative abundance of individual genera and AR (FDR adjusted *P* = 0.45 for *Bifidobacterium* and *P* = 0.37 for *Prevotella*).

The second composite variable calculated from NMF, which was positively correlated with the relative abundance of *Bifidobacterium*, was also associated with the intake of several nutrients, including carbohydrate [CHO], saturated fat [SFA], and alcohol [ALC], and the latter two nutrients were also associated with the third composite variable (Fig. 4i). CHO, SFA, and ALC were also associated with the second composite variables calculated using the JSD (Fig. 4a,f) and BCD (Fig. 4b,g). The third composite variable calculated by PCoA with JSD was positively associated with nutrient intakes including dietary fibre (total dietary fibre [TDF], insoluble dietary fibre [NDF], and soluble dietary fibre [WDF]), magnesium [MG], potassium [K], niacin [NAC], thiamin [VB1], and vitamin B6 [VB6] (Fig. 4a). The composite variables calculated by PCA were not associated with any nutrient and AR (FDR adjusted *P* value > 0.05) (Fig. 4d), while the second composite variable calculated by PCA of CLR-transformed abundance data was negatively associated with ALC (Fig. 4e).

## Discussion

Dimension reduction has been used to visualise the distribution of multidimensional microbiome data^11,12^, but the composite variables calculated by the dimension reduction methods have not been widely used to investigate the relationship of the human gut microbiome with lifestyle and disease. In the present study, we applied several dimension reduction methods, including PCA, PCoA, NMDS, and NMF, to a microbiome dataset from 186 subjects with symptoms of AR and 106 controls. All the dimension reduction methods supported that the microbial composition appeared to be continuous rather than discrete. The top 3 composite variables obtained from JSD and BCD were highly correlated with each other, and those obtained from the UniFrac distance were also highly correlated with each other, whereas the top 3 composite variables from other methods did not correspond, suggesting that the characteristics of the composite variables differed depending on the distance matrices and the dimension reduction methods. We found that the first composite variables calculated from PCoA and NMDS with the UniFrac distance were strongly associated with AR, and also with the relative abundance of *Bifidobacterium* and *Prevotella*. Unlike JSD and BCD, which focus primarily on differences in abundance or composition without considering phylogeny, UniFrac incorporates both the relative abundance of bacteria and their phylogenetic relationships^24^. Another difference is that JSD and BCD were calculated from the genus-level abundance data in our study, whereas the weighted UniFrac distance used classification at the operational taxonomic unit (OTU) level. The variability in the strength of the association between AR and composite variables across different distances and dimension reduction methods underscores the importance of using a variety of analytical approaches to uncover the complex interplay between the microbial community and human health.

The genus *Bifidobacterium* has been recognized to confer health benefits to the host also through its interaction with the host’s immune system^34,35^. These benefits comprise both local effects, which result from the contribution of *Bifidobacterium* to the intestinal barrier function—which ultimately translates into systemic health—and systemic effects, which stem from the microorganism’s impact on specific pathways through extracellular structures and metabolites. An example of this is the anti-inflammatory response that is elicited by acetate produced by *Bifidobacterium*^36^. *Bifidobacterium* can digest complex carbohydrates, such as glucans, into acetate which is further digested into butyrate by other gut microorganisms. Butyrate is known to possess anti-inflammatory properties that include the production of TGF-β, IL-18, and IL-10 cytokines by antigen-presenting cells and IECs, which together stimulate the differentiation of naïve T cells into Treg cells. Additionally, *Bifidobacterium* produce two types of pili, hair-like structures found on the surface of bacteria, and pili produced in certain bifidobacterial strains have been shown to stimulate TNF-α levels in macrophages while suppressing other pro-inflammatory cytokines that are associated with systemic immune responses^37^.

The association between *Bifidobacterium* and allergic symptoms has indeed been reported. For example, an observational microbiome study reported that patients with atopy and asthma tended to have a lower abundance of *Bifidobacterium*^38^. Although studies on the relationship between allergic rhinitis and the intestinal *Bifidobacterium* abundance are limited, one study reported that the symptom of allergic rhinitis was reduced by oral administration of probiotic *B. lactis*^39^. However, the increase in intestinal *Bifidobacterium* was not confirmed. In addition, it is debated whether *Bifidobacterium* is beneficial or not, and at least, the genus *Bifidobacterium* is not uniformly beneficial^40^.

The abundance of *Bifidobacterium* was further linked to increased intakes of carbohydrate and decreased alcohol consumption by several dimension reduction methods, including PCoA and NMDS using JSD and BCD, and NMF. Consumption of non-digestible carbohydrates has been shown to promote the growth of *Bifidobacterium*^41^. Excessive alcohol consumption has been shown to lead to an imbalance in the gut environment and a reduction in *Bifidobacterium* populations^42^.

There are several limitations to this study. First, we did not evaluate the performance of the different dimension reduction methods. We applied several dimension reduction methods to a case–control dataset and examined the association of the composite variables calculated using different dimension reduction methods with nutrient intake and AR. Further studies are needed to investigate whether dimension reduction methods can contribute to an increased ability to distinguish patients from controls on the basis of discriminatory power. Second, we only analysed one microbiome dataset. Further research using a variety of microbiome datasets is warranted to determine the advantages and disadvantages of dimension reduction methods. For example, our data showed that the first composite variable calculated by NMF was positively correlated with the P/B ratio, suggesting that NMF may be able to extract a meaningful dimension from microbiome data alone without prior knowledge.

In conclusion, our results highlight the usefulness of the dimension reduction methods for investigating the association of microbial composition with lifestyle and disease in clinical research.

## Data Availability

The data are not available for public access because of participant privacy concerns, but are available from the corresponding author on reasonable request.
